# Glycated Haemoglobin Versus Serum Fructosamine in Assessing Glycaemic Control Amongst Type 2 Diabetes Mellitus Patients With Haemoglobin Variants: A Systematic Review

**DOI:** 10.1155/bmri/9298499

**Published:** 2026-11-14

**Authors:** Edward Morkporkpor Adela, Richard Kojo Yankah, George Nkrumah Osei, Wisdom Agbotey, Patrick Adu

**Affiliations:** ^1^ Department of Medical Laboratory Science School of Allied Health Sciences University of Cape Coast Cape Coast Ghana ucc.edu.gh; ^2^ Department of Family Medicine Cape Coast Teaching Hospital Cape Coast Ghana ccthghana.org; ^3^ Department of Immunology and Microbiology School of Medical Sciences University of Cape Coast Cape Coast Ghana ucc.edu.gh

**Keywords:** fructosamine, glycaemic monitoring, glycated albumin, haemoglobin variants, HbA1c, Type 2 diabetes mellitus

## Abstract

Haemoglobin variants lead to shortened red cell lifespan and may affect the reliability of HbA1c. This review synthesised evidence on the validity, reliability and practical utility of HbA1c versus serum fructosamine (SF) in Type 2 diabetes mellitus (T2DM) adults with haemoglobinopathies and mapped assay‐ and phenotype‐specific interferences. A systematic review was conducted on articles indexed in PubMed, Ovid MEDLINE and Scopus following PRISMA 2020 guidelines. The protocol was prospectively registered on PROSPERO (ID: CRD420251128494). Seventeen studies out of 85 records met the inclusion criteria. HbA1c appeared to remain valid in heterozygous carriers but was unreliable in individuals with homozygous or compound variants, particularly when measured using ion‐exchange HPLC. SF may provide an alternative measure of glycaemic status when HbA1c was invalidated by haemoglobin variants. Large cohort evidence showed that fructosamine and glycated albumin maintained strong correlations with fasting glucose and continuous glucose monitoring (CGM) indices, achieving high diagnostic accuracy for uncontrolled glycaemia. HbA1c appears appropriate for most heterozygous haemoglobinopathy carriers using NGSP/IFCC‐aligned assays, whereas fructosamine and glycated albumin could be reliable short‐term alternatives in homozygous or compound variants, contingent on albumin status. Standardised, multicentre accuracy studies across assay families and genotypes are needed to refine guidance.

## 1. Background

Diabetes mellitus (DM) represents a major global health challenge, affecting millions of individuals and contributing significantly to morbidity and mortality. Globally, an estimated 589 million adults (aged 20–79 years) were living with DM in 2024, a figure projected by the International Diabetes Federation to increase to 853 million by 2050 [1]. Type 2 diabetes mellitus (T2DM) accounts for the vast majority of adult DM cases, commonly estimated at ~90%–96% in recent global analyses [2, 3], implying roughly 530–565 million adults with T2DM in 2024, and projected to rise to 768–819 million by 2050. Additionally, conservative projections indicate that several hundred million adults with T2DM will require diagnosis and long‐term management in the coming decades. DM is amongst the leading causes of death worldwide and is a major contributor to blindness, kidney failure, heart attacks, stroke and lower extremity amputation [4]. Much of this burden can be mitigated through evidence‐based interventions, including accurate monitoring of glycaemic control. The World Health Organisation emphasises that robust DM monitoring indicators are critical for guiding prevention and treatment programmes [4].

Glycaemic monitoring plays a central role in the management of T2DM, enabling clinicians to adjust therapy and prevent complications. Glycated haemoglobin (HbA1c) reflects average blood glucose levels over approximately 3 months and has become a cornerstone for diagnosing DM and assessing long‐term glycaemic control. The HbA1c test measures glucose attached to haemoglobin in red blood cells, with higher glucose levels resulting in higher HbA1c values [5]. Despite the widespread use of HbA1c, several conditions including sickle cell anaemia, thalassaemia, kidney disease, liver disease and blood transfusion interfere with its accuracy [6]. These conditions can alter red blood cell survival, leading to falsely low or high HbA1c values.

Haemoglobin variants are particularly problematic. Haemoglobin S, C, E and D, as well as elevated foetal haemoglobin (HbF), may cause clinically significant interference in some HbA1c methods [5]. Charge‐based HbA1c methods such as high‐performance liquid chromatography (HPLC) and capillary electrophoresis separate haemoglobin species by charge and are susceptible to interference from nonglucose adducts such as carbamylation in uraemia and structural changes in haemoglobin variants [7]. More than 1000 human haemoglobin variants have been described, and depending on their charge, these variants may migrate or coelute with HbA1c or HbA, causing incorrect interpretation [7]. Immunoassays target the N‐terminal glycated amino acids of the *β* chain; any amino acid substitution in this region, such as in Hb S or Hb C, can hinder antibody binding and produce inaccurate HbA1c values [8]. Patients with high levels of HbF (> 10%) may also experience falsely low HbA1c results because HbF lacks the *β* chain targeted by most assays [9]. Although boronate affinity chromatography specifically measures glycated fractions and is largely free from haemoglobin variant interference, rare variants with excessive glycation can still cause spurious results. Importantly, in individuals with homozygous or compound heterozygous variants (e.g. Hb SS, Hb CC and Hb SC), HbA1c is absent because there is no HbA, rendering HbA1c testing invalid [7].

In settings where HbA1c is unreliable, alternative biomarkers such as serum fructosamine and glycated albumin may offer advantages. Serum fructosamine reflects the nonenzymatic glycation of circulating proteins, mainly albumin, globulins and lipoproteins. Because serum proteins have a shorter half‐life (14–21 days), serum fructosamine provides a 2–3‐week snapshot of glycaemic control [10]. Unlike HbA1c, serum fructosamine and glycated albumin are unaffected by haemoglobin level or red blood cell characteristics, making them potential candidates for glycaemic monitoring of T2DM patients with conditions such as haemoglobinopathies, sickle cell anaemia and other anaemias.

Overall, Africa, South Asia and the Middle East represent high prevalence of haemoglobinopathies, both in terms of carrier frequency and annual affected births [11]. Recent global estimates also show a substantial rise in the burden of sickle cell disease. In 2021, about 7.74 million people were living with SCD worldwide, an increase from 5.46 million in 2000. In the same year, there were roughly 515,000 new SCD births, with about one‐fifth of these occurring in sub‐Saharan Africa [12]. Furthermore, according to the International Diabetes Federation, 1 in 20 adults in Africa (≈25 million) currently live with diabetes. The number of adults with diabetes in this region is projected to increase by 142% to around 60 million by 2050, and many remain undiagnosed [1]. In the Middle East and North Africa, one in six adults (≈85 million) has diabetes, the highest regional proportion; the burden is expected to increase by 92% to ~163 million by 2050 [1]. Such statistics underscore the twin challenges of rising diabetes prevalence and high rates of haemoglobinopathies in sub‐Saharan Africa and parts of Asia, motivating the need for robust glycaemic markers beyond HbA1c. Despite growing awareness of assay interference, existing literature is limited, consisting mostly of case reports, and lacks a systematic comparison of HbA1c with alternative biomarkers such as serum fructosamine and glycated albumin across diverse haemoglobin phenotypes.

This systematic review therefore is aimed at evaluating how HbA1c compares to serum fructosamine and/or glycated albumin when determining glycaemic control amongst adults diagnosed with T2DM who have haemoglobin variants. Secondary goals include the assessment of interference from assays, the effect of haemoglobin genotype and the clinical implications of discordant glycaemic marker results.

## 2. Methods

### 2.1. Protocol and Registration

The review adhered to the Preferred Reporting Items for Systematic Reviews and Meta‐Analyses (PRISMA) 2020 guidelines [13]. The protocol was registered prospectively with PROSPERO (ID: CRD420251128494) before literature searches were initiated in September 2025. Ethical approval was not required because data were derived from published studies.

### 2.2. Eligibility Criteria

The review included (1) original, peer‐reviewed randomised controlled trials, cohort or cross‐sectional studies, case series and case reports involving human participants diagnosed with T2DM and reporting inherited haemoglobin variants; (2) studies that explicitly reported participants with inherited haemoglobin variants; (3) studies reporting of fasting blood glucose, HbA1c and/or serum fructosamine or glycated albumin measurements; (4) studies assessing the accuracy, reliability or validity of HbA1c and/or serum fructosamine in glycaemic monitoring and (5) studies published in English. Studies involving Type 1 DM, gestational diabetes or cell line models of T2DM or nonhuman models of DM were excluded. Reviews, opinion papers, editorials, commentaries and abstracts without full texts were excluded. Case reports were included due to the rarity of certain haemoglobin variants. Studies published in non‐English languages were excluded due to the non‐English language competencies of the research team.

### 2.3. Search Strategy

Database searches were performed in PubMed, Ovid MEDLINE and Scopus from 2 September 2025 to 31 October 2025. The search combined MeSH terms and key terms related to glycaemic biomarkers (‘hemoglobin A1c’, HbA1c, ‘glycated hemoglobin’, fructosamine and ‘glycated albumin’), haemoglobin variants (Hb AS, Hb SC, Hb CC, Hb SS, ‘hemoglobin variant’ and sickle) and disease context (‘type 2 diabetes’ and T2DM) to identify studies comparing biomarker performance across diverse haemoglobin phenotypes. The detailed search strategies for all databases are provided in Supporting Information 1: File S1. Reference lists of included articles and relevant reviews were hand‐searched. No date or geographical restrictions were applied.

### 2.4. Selection Process

Titles and abstracts identified by the search were imported into Mendeley Reference Manager, and duplicates were removed. Two reviewers (E.M.A. and W.A.) independently screened titles and abstracts against the eligibility criteria. The searches retrieved 126 records (PubMed, *n* = 31; Ovid MEDLINE, *n* = 25; Scopus, *n* = 70). After removal of 41 duplicate records across the combined database results, 85 unique records were screened, of which 21 full‐text reports were assessed for eligibility (Figure 1). Four full‐text reports were excluded: One lacked an appropriate index test, two did not involve T2DM and one [14] enrolled mixed Type 1 and Type 2 diabetes populations without separately extractable T2DM‐specific outcome data. Seventeen studies were therefore included in the review. Discrepancies were resolved through discussion with a third reviewer (G.N.O.).

**Figure 1:**
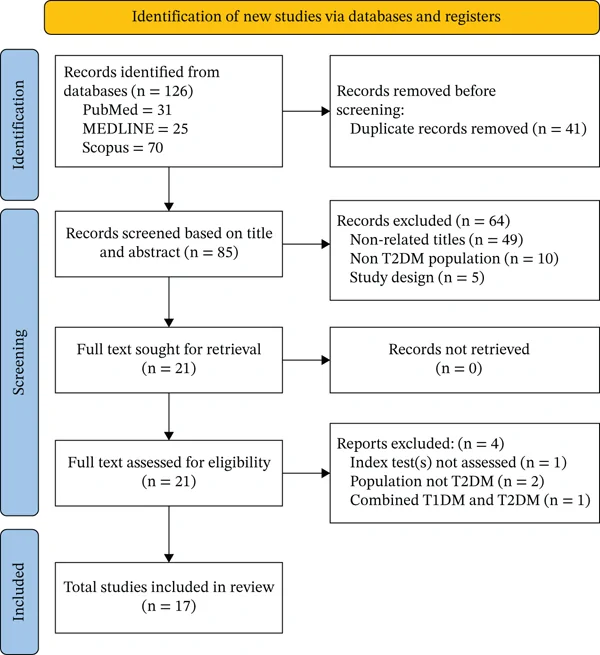
PRISMA flowchart illustrating the identification, screening and selection processes for studies included in the review. The flow diagram demonstrates the study selection process for the review. The search retrieved 126 records (PubMed = 31, Scopus = 70 and MEDLINE = 25). After removing 41 duplicates, 85 titles and abstracts were screened, of which 21 full texts were assessed for eligibility. Four full texts were excluded (one lacked an appropriate index test, two did not involve T2DM and one combined T1DM and T2DM patients), leaving 17 studies for inclusion in the final review. T1DM: Type 1 diabetes mellitus; T2DM: Type 2 diabetes mellitus.

### 2.5. Data Extraction

A standardised data extraction form was used to extract data on study characteristics (author, year, country and design), participant demographics (sample size, age, sex and phenotype), HbA1c and/or serum fructosamine assay methods (platform, NGSP [National Glycohaemoglobin Standardisation Program]/IFCC [International Federation of Clinical Chemistry and Laboratory Medicine] alignment), comparator and context of fasting blood glucose (laboratory measurement, oral glucose tolerance test and self‐monitoring or continuous glucose monitoring), outcome types (correlation coefficients, Bland–Altman bias and diagnostic accuracy metrics), key numerical findings and comments on assay interference. This was done by two authors (E.M.A. and W.A.), and any discrepancies were resolved by consensus through discussion. When studies reported multiple assays or phenotypes, data were extracted separately for each subgroup.

The extracted data from the 17 included studies are summarised in Table 1.

**Table 1:** Summary of key characteristics and findings from the included studies.

**S/N**	**Authors (year), country**	**Design**	**Population/variant breakdown**	**Assays (HbA1c and serum fructosamine platforms; NGSP/IFCC alignment)**	**Comparator and FBG context**	**Outcome types**	**Key numerical findings**	**Notes**
1	Niwaha et al. (2021), Uganda [15]	Multicentre observational cohort	192 adults with T2DM; approximately 33% of participants had at least one of the following: Anaemia, a haemoglobin variant or renal impairment (exact distribution not reported) Hb AS present but not numerically disaggregated)	HbA1c by latex agglutination immunoassay (Roche Cobas 6000, IFCC aligned); serum fructosamine by enzymatic assay (Cobas 8000); glycated albumin by Lucica GA‐L; haemoglobin variants detected via capillary electrophoresis	CGM mean glucose over 14 days; FBG measured at baseline visit using glucokinase method	Correlation, Bland–Altman, diagnostic accuracy	HbA1c vs. CGM *r* = 0.88; serum fructosamine vs. CGM *r* = 0.84; GA vs. CGM *r* = 0.84; FBG vs. CGM *r* = 0.97; FBG threshold 7.1 mmol L^−1^ predicted HbA1c ≥ 7% (sensitivity 78.2%, specificity 78.8%)	Anaemia and sickle cell trait caused modest underestimation of CGM glucose by HbA1c and fructosamine; GA less affected
2	Niwaha et al. (2025), Uganda [16]	Method comparison analysis of the same OPTIMAL cohort: A reanalysis of data collected in 2021, assessing additional biomarkers (serum fructosamine and glycated albumin)	192 adults with T2DM; ~33% had anaemia, haemoglobin variants or renal impairment (exact variant distribution not reported separately; Hb AS present but not numerically disaggregated)	HbA1c by latex agglutination immunoassay (Roche Cobas 6000, IFCC aligned); serum fructosamine by enzymatic assay (Cobas 8000); glycated albumin by Lucica GA‐L; haemoglobin variants detected via capillary electrophoresis	CGM mean glucose; FBG measured at subsequent visit	Correlation and regression; Bland–Altman	HbA1c and serum fructosamine underestimated CGM mean glucose in anaemic participants; GA correlation unaffected by sickle cell trait	Highlights GA′s robustness
3	Skinner et al. (2019), Senegal [17]	Analytical cross‐sectional study	203 adults (97 with T2DM); 25.6% (≈52) had Hb AS	HbA1c by capillary electrophoresis (Sebia CAPILLARY 3 Tera; NGSP/IFCC certified); serum fructosamine by nitroblue tetrazolium assay; FBG by glucose oxidase	Fasting blood sample after overnight fast	Correlations; Bland–Altman	In Hb AA participants, HbA1c vs. FBG *r* = 0.32 and serum fructosamine vs. FBG *r* = 0.31; in Hb AS participants, HbA1c vs. FBG *r* = 0.53 and serum fructosamine vs. FBG *r* = 0.58 Bland–Altman bias showed FBG values 5.54*%* ± 45.7*%* higher than HbA1c in Hb AA and 12.2*%* ± 52.9*%* higher in Hb AS	Demonstrated poor interchangeability between markers; emphasised need to use same assay when monitoring a patient
4	Doumatey et al. (2021), Nigeria [18]	Analytical cross‐sectional study with validation cohort	Primary cohort (*n* = 618): 286 with T2DM; ~25% (≈155) had Hb AS. Validation cohort (*n* = 634): Similar ~25% Hb AS distribution	HbA1c by latex agglutination immunoassay on DCA Vantage (NGSP/IFCC); serum fructosamine by nitroblue tetrazolium on Roche Modular E; FBG and 2‐h OGTT by glucose oxidase; haemoglobin variants by cellulose acetate electrophoresis at alkaline pH	Comparator: HbA1c < 7% or FBG < 130 mg dL^−1^ (controlled) vs. uncontrolled; FBG sample at baseline; 2‐h OGTT used for reference interval but not for main comparisons	Correlations; AUROC; sensitivity/specificity	Serum fructosamine correlated with HbA1c (*r* = 0.80 for Hb AA and *r* = 0.82 for Hb AS) and FBG (*r* = 0.72 and 0.83); AUC for serum fructosamine in detecting uncontrolled glycaemia 0.88–0.92; cutoff 352.5 *μ*mol L^−1^ yielded sensitivity 81.5% and specificity 97.4%; for diagnosing T2DM, the cutoff 309.5 *μ*mol L^−1^ gave sensitivity 82.4% and specificity 92.4%	Hb AS had no effect on serum fructosamine performance
5	Ama et al. (2012), Cameroon [19]	Cross‐sectional study	73 adults with T2DM; 19% (≈14) had Hb AS; plus 40 nondiabetic controls	HbA1c by ion‐exchange HPLC (Human Diagnostics kits); FBG by glucose oxidase; haemoglobin phenotype by alkaline cellulose acetate electrophoresis	Fasting plasma glucose from baseline laboratory sample	Regression and correlation	Hb AS unrelated to fasting glucose (*β* = 0.02; 95% CI −37.68 to 43.30) and HbA1c (*β* = −0.03; 95% CI −1.18 to 0.93); Hb AS did not affect the relationship between FBG and HbA1c	Suggested sickle cell trait does not influence metabolic control; small sample
6	Kosecki et al. (2005), United States [20]	Case report	Single case: Woman with T2DM + Hb S/*β*‐thalassaemia compound	HbA1c by unspecified immunoassay; serum fructosamine by nitroblue tetrazolium assay	Comparator: SMBG logs (fasting 170–320 mg dL^−1^); no laboratory FBG or OGTT	Narrative comparison	Despite high SMBG values, HbA1c remained < 7%; serum fructosamine was 340 *μ*mol L^−1^, indicating poor glycaemic control	Proposed serum fructosamine as more appropriate than HbA1c in compound haemoglobinopathies
7	Thevarajah et al. (2009), Malaysia [21]	Case report	Single case: Woman with T2DM + heterozygous Hb D Punjab	HbA1c by immunoturbidimetry (Cobas Integra) and ion‐exchange HPLC (ARKRAY HA8160)	FBG at baseline laboratory sample (6.2–7.8 mmol L^−1^)	Narrative comparison	Immunoturbidimetry produced HbA1c 6.11%–7.23%, whereas ion‐exchange HPLC failed to detect HbA1c	Showed that immunoassay may be reliable when HPLC fails
8	Castelli et al. (2004), Italy [22]	Case report	Single case: Man with T2DM + Camperdown haemoglobin variant	HbA1c by cation‐exchange HPLC (Bio‐Rad VARIANT HbA1c Programme); haemoglobin variant identified by reverse‐phase HPLC and DNA sequencing	Fasting plasma glucose 18.04 mmol L^−1^	Narrative	CE‐HPLC chromatogram showed abnormal peak; HbA1c could not be reliably quantified; DNA sequencing confirmed *β*‐globin mutation	Emphasised need to inspect chromatograms and perform confirmatory testing
9	Yuan et al. (2020), China [23]	Case report	Single case: Man with T2DM + Hb North Manchester	HbA1c measured via ion‐exchange HPLC (Tosoh HLC‐723 G8 and Bio‐Rad VARIANT II), capillary electrophoresis (Sebia CAPILLARY 2 Flex Piercing), boronate affinity HPLC (Premier Hb9210) and glycated albumin immunoassay; variant sequencing by Sanger	Comparator: Home SMBG (fasting 6.8–7.7 mmol L^−1^; 2‐h postprandial 7.1–7.5 mmol L^−1^) and fasting plasma glucose 5.97 mmol L^−1^	Narrative	Ion‐exchange HPLC gave low HbA1c (4.3%–5.5%) despite moderate glucose levels; glycated albumin 12.45% (normal range 11%–16%); capillary electrophoresis showed variant peaks 0.4%–5.5%	Demonstrated that some variants produce discordant HbA1c results even when other assays appear normal
10	Sadriani and Marpaung (2023), Indonesia [24]	Case report	Single case: Woman with T2DM + homozygous Hb E	HbA1c by turbidimetric immunoassay (51 mmol mol^−1^); ion‐exchange HPLC (Bio‐Rad D‐10) yielded no HbA1c peak; glycated albumin 3.1 mmol L^−1^	FBG (mean 7.7 mmol L^−1^) and 2‐h plasma glucose (mean 13 mmol L^−1^)	Narrative	Turbidimetric HbA1c appeared moderately elevated, but ion‐exchange HPLC showed ‘no result’; glycated albumin elevated; capillary electrophoresis confirmed homozygous Hb E	Suggested that glycated albumin is useful when HPLC fails to detect HbA1c
11	Danescu et al. (2010), United States [25]	Case report	Single case: Man with T2DM + *β*‐thalassaemia minor	HbA1c method not reported; red cell survival measured by chromium‐51; serum fructosamine proposed	FBG from repeated laboratory tests; OGTT used for diagnosis	Narrative	HbA1c extremely low (1.6%–3%) despite fasting glucose > 11.1 mmol L^−1^; red cell lifespan < 25% of normal	Highlighted that HbA1c is unreliable in *β*‐thalassaemia; serum fructosamine recommended
12	Takeda et al. (2015), Japan [26]	Case report	Single case: African man with T2DM + Hb S trait	HbA1c by immunonephelometry (CHM 4100) and ion‐exchange HPLC (Tosoh HLC‐723 G7); glycoalbumin measured (method not reported)	Casual plasma glucose 536 mg dL^−1^ (nonfasting)	Narrative	Immunonephelometric HbA1c 13.3%; HPLC HbA1c 7.6%; glycoalbumin 47.5%; casual glucose 536 mg dL^−1^	Demonstrated that immunoassay may give higher HbA1c than HPLC in Hb S trait
13	Karki et al. (2024), Nepal [27]	Case report	Single case: Woman with T2DM + homozygous Hb E	HbA1c by ion‐exchange HPLC (Bio‐Rad VARIANT II) and Tosoh 7234X (both NGSP aligned); haemoglobin capillary electrophoresis for confirmation	FBG 173 mg dL^−1^; postprandial 280 mg dL^−1^	Narrative	HbA1c values extremely low (1.1% on VARIANT II; 2.1% on Tosoh) despite high fasting and postprandial glucose	Highlighted that homozygous Hb E causes spurious HbA1c results; recommended boronate affinity or alternative markers
14	Guedes et al. (2017), Portugal [28]	Case report	Single case: Woman with T2DM + Hb Himeji variant	HbA1c by ion‐exchange HPLC (Bio‐Rad platform cited in text); capillary electrophoresis (Sebia system); serum fructosamine not reported	FBG assessed as baseline laboratory fasting draw; no OGTT/CGM	Narrative discordance; method interference	FPG 190 mg/dL; HbA1c 4.1%; repeat HbA1c 4.5% by HPLC	Manual review of cation‐exchange chromatograms prompted variant workup
15	Cheng et al. (2015), China [29]	Case report	Single case: Woman with T2DM + Hb G‐Coushatta	HbA1c by CE‐HPLC with interfering peak; haemoglobin profiling by capillary electrophoresis; glycated albumin measured; immunoassay mentioned (platform not specified)	FBG assessed as baseline laboratory fasting draw; no OGTT/CGM	Narrative interference; discordance; use of GA	Elevated fasting blood glucose; precise HbA1c/GA values not reported in extract	DNA sequencing of HBB confirmed Hb G‐Coushatta; CE flagged variant
16	Yordanov et al. (2020), Bulgaria [30]	Case report	Single case: Adult with T2DM + *β*‐thalassaemia minor	HbA1c platform not reported; haemoglobin evaluation includes capillary‐based methods; serum fructosamine not reported	FBG assessed as baseline laboratory fasting draw; OGTT performed; no CGM	Narrative unreliability of HbA1c; interpretive guidance	Specific numerical accuracy statistics not reported	Argues for privileging glucose‐based comparators when HbA1c is discordant
17	Dholariya et al. (2024), India (Gujarat) [31]	Case report (method‐focused detection)	Single case: Adult with T2DM + Hb D Punjab trait	HbA1c/variant detection by ion‐exchange HPLC; platform not specified; serum fructosamine not reported	FBG assessed as baseline laboratory fasting draw (133 mg/dL)	Narrative interference; chromatographic separation issue	Discordant HbA1c relative to FBG; no correlation/AUC reported	Emphasises inspection of chromatograms when clinical laboratory discordance exists

Abbreviations: *β*, regression coefficient; AUROC/AUC, area under the receiver operating characteristic curve; CE, capillary electrophoresis; CGM, continuous glucose monitoring; CI, confidence interval; FBG, fasting blood glucose; FPG, fasting plasma glucose; GA, glycated albumin; Hb, haemoglobin; HbA1c, glycated haemoglobin; Hb AA, normal adult haemoglobin phenotype; Hb AS, sickle cell trait; Hb D/Hb D Punjab, haemoglobin D variant; Hb E, haemoglobin E variant; HPLC, high‐performance liquid chromatography; IFCC, International Federation of Clinical Chemistry and Laboratory Medicine; mg/dL, milligramme per decilitre; NGSP, National Glycohaemoglobin Standardisation Program; OGTT, oral glucose tolerance test; SMBG, self‐monitoring of blood glucose; T2DM, Type 2 diabetes mellitus.

### 2.6. Quality Assessment

Methodological quality of the 17 included studies was assessed independently by two reviewers (E.M.A. and W.A.) using the Joanna Briggs Institute (JBI) critical appraisal tool appropriate to each study design. JBI appraisal was used to evaluate risk of bias at the individual study level. The detailed study‐level appraisal is presented in Supporting Information 1: File S2. Discrepancies were resolved through discussion and, where necessary, involvement of a third member of the research team. JBI judgements were not used as certainty‐of‐evidence ratings.

### 2.7. Certainty of Evidence (Grading of Recommendations Assessment, Development and Evaluation [GRADE])

Certainty of the body of evidence was assessed at the outcome level using the GRADE framework. The domains considered were study limitations, inconsistency, indirectness, imprecision and publication bias. GRADE ratings were assigned to outcome‐level bodies of evidence rather than to individual studies. Since Niwaha et al. and Niwaha et al. analysed the same OPTIMAL cohort, the two reports were treated as one underlying dataset when judging certainty [15, 16]. Outcome‐level certainty ranged from low to moderate (Table 2). Evidence concerning HbA1c performance in common heterozygous variants was rated moderate because a fair number of observational datasets were available. Evidence for serum fructosamine and glycated albumin was rated low because only a few independent datasets were available and methods varied across studies. Evidence concerning severe HbA1c discordance in homozygous, compound or rare variants, and evidence concerning assay‐specific interference, was rated low because it was derived predominantly from case reports and was affected by serious imprecision and likely publication or rarity bias. The resulting GRADE summary of findings is presented in Table 2.

**Table 2:** Grading of Recommendations Assessment, Development and Evaluation (GRADE) summary of findings.

**Outcome**	**Evidence base and principal finding**	**Main GRADE considerations**	**Certainty**
HbA1c performance in common heterozygous variants	Four independent observational datasets (five reports; the two Niwaha reports analysed the same OPTIMAL cohort) generally preserved an association between HbA1c and glucose‐based measures in common heterozygous variants, especially Hb AS, although effect magnitude varied by assay and comparator	Some variant subgroups were incompletely disaggregated; assay platforms and glycaemic comparators differed; smaller studies were imprecise	Moderate
Serum fructosamine as an alternative glycaemic marker	Three independent observational datasets plus supporting case reports showed correlations with FBG, CGM or HbA1c. Doumatey et al. reported AUROC values of 0.88–0.92 for uncontrolled glycaemia	Few independent datasets; cross‐sectional evidence; fructosamine methods and cutoffs varied; limited variant‐specific precision	Low
Glycated albumin as an alternative glycaemic marker	One independent cohort reported in two analyses, supported by several case reports, showed correlation with CGM and potential usefulness when HbA1c was discordant	Very limited independent evidence; incomplete phenotype disaggregation; indirectness and imprecision; possible publication bias	Low
Severe HbA1c discordance in homozygous, compound or rare variants	Predominantly single‐patient case reports described markedly discordant or unquantifiable HbA1c in selected haemoglobin variants	No denominator or frequency estimates; serious imprecision; selection, rarity and publication bias; limited generalisability	Low
Assay‐specific haemoglobin variant interference	Case reports using HPLC, immunoassay, capillary electrophoresis or boronate affinity methods indicated that interference depends on the specific variant assay combination	Sparse head‐to‐head comparisons; heterogeneous platforms and variants; selective reporting of unusual assay failures	Low

*Note:* Certainty of the body of evidence was assessed at the outcome level using the Grading of Recommendations Assessment, Development and Evaluation (GRADE) framework. Overall certainty was rated as low to moderate.

### 2.8. Data Synthesis

Heterogeneity was assessed qualitatively by comparing study design, haemoglobin type, HbA1c assay platform, alternative biomarker method, glycaemic comparator and reported effect measure. Formal meta‐analysis was limited because the included studies did not provide a sufficient set of clinically comparable, independent estimates for a common outcome. Twelve of the 17 included studies were single‐patient case reports and therefore did not provide group‐level effect estimates or variance measures suitable for pooling. The remaining five observational reports differed in assay methods, haemoglobin variants, glycaemic comparators (including fasting glucose, CGM, self‐monitoring blood glucose [SMBG] and OGTT) and statistical outputs (correlations, regression estimates, Bland–Altman measures, diagnostic accuracy statistics or narrative assay discordance). In addition, two reports analysed the same OPTIMAL cohort and were not statistically independent. Only one independent study reported formal diagnostic accuracy estimates for serum fructosamine, precluding a diagnostic accuracy meta‐analysis. Because no clinically interpretable common effect estimate could be defined, statistical heterogeneity statistics such as *I* ^2^ were not calculated. Findings were therefore synthesised narratively.

## 3. Results

### 3.1. Characteristics of Included Studies and Participants

The 17 included reports were published between 2004 and 2025 and originated from Africa (*n* = 5; Uganda = 2, Senegal = 1, Cameroon = 1 and Nigeria = 1), Asia (*n* = 7; China = 2, India = 1, Indonesia = 1, Japan = 1, Malaysia = 1 and Nepal = 1), Europe (*n* = 3; Portugal = 1, Italy = 1 and Bulgaria = 1) and North America (*n* = 2; United States = 2). Five reports used non–case report observational designs: Two reports analysed the same multicentre OPTIMAL cohort (one primary analysis and one reanalysis), and three were cross‐sectional studies; the remaining 12 reports [20–31] were single‐patient case reports. Because the two Niwaha reports analysed the same 192‐participant cohort and the cross‐sectional studies included nondiabetic comparison groups, a single aggregate participant total was not reported in order to avoid double‐counting overlapping participants or combining eligible T2DM participants with comparison participants. Haemoglobin phenotypes comprised Hb S (including sickle cell trait, Hb AS), Hb AC trait, Hb E (homozygous Hb EE), Hb North Manchester, Hb Camperdown (*β*104[G6] Arg → Ser), Hb G‐Coushatta, *β*‐thalassaemia minor and the compound state sickle cell plus *β* thalassaemia. Participants were predominantly adults with T2DM; three cross‐sectional studies [17–19] enrolled both T2DM patients and nondiabetic controls.

The main HbA1c assay platforms were ion‐exchange HPLC (Tosoh G8, Bio‐Rad VARIANT II), capillary electrophoresis (Sebia CAPILLARY 3 TERA), boronate affinity chromatography (Trinity Biotech, ARKRAY), immunoassays (latex agglutination, turbidimetric and enzymatic) and enzymatic assays (Roche Cobas). NGSP or IFCC alignment was reported in 12 studies. Serum fructosamine was measured using the nitroblue tetrazolium reduction method in eight studies and by enzymatic assays in five studies. Glycated albumin was measured by the Lucica GA‐L enzymatic assay in two studies. Capillary electrophoresis, chromatography or high‐resolution HPLC was used to detect haemoglobin variants in most studies.

### 3.2. Risk of Bias Integration

Risk of bias was assessed qualitatively across study designs (Table 1). The Ugandan and Nigerian cohorts were prospective and used NGSP/IFCC‐aligned assays, but they could not fully control for confounding from anaemia, renal impairment and medication use. The Senegalese study had a modest sample size and cross‐sectional design, limiting causal inference. The Cameroonian study included only 73 patients and reported wide confidence intervals around regression coefficients. All other evidence arose from case reports or method‐comparison papers without control groups, carrying a high risk of selection bias and confounding.

## 4. Discussion

Haemoglobin variants have been linked with HbA1c measurement interferences. In a cohort study of African patients, Hb AS and Hb AC showed strong correlations between certified HbA1c values and average glycaemia [15, 18]. These align with NGSP/IFCC guidance, which notes that appropriately chosen methods yield reliable HbA1c values in trait carriers [5]. Conversely, other reports documented inaccurately low or unquantifiable HbA1c values in individuals with homozygous or compound heterozygous mutations affecting all *β*‐globin genes (such as Hb SS, Hb CC, Hb SC or Hb EE), especially on charge‐based ion‐exchange HPLC [22, 27, 29]. This concurs with Association for Diagnostics and Laboratory Medicine (ADLM) commentary that patients without normal HbA ‘do not produce HbA1c’ [7].

Some striking mismatches between HbA1c and contemporaneous glucose measurements include the observation of near‐normal or extremely low HbA1c in patients with rare *β*‐chain variants despite clearly elevated glucose [27], as well as reports of overestimation [26]. These discrepancies mirror the observations of previous researchers who noted that haemoglobin variants can produce falsely low or high HbA1c results [6], depending on assay type [31], underscoring the limitations of relying solely on a single HbA1c measurement when discordant with concurrent glucose assessments.

Analytical interference occurs when variant haemoglobin affects HbA1c measurement. Ion‐exchange HPLC has been most frequently associated with spurious results. Charge‐based methods are most vulnerable to interference, whereas boronate affinity and immunoassays are relatively accurate [5, 32]. This review observed that ion‐exchange HPLC yielded spuriously low or unquantifiable HbA1c values in cases involving Hb E homozygote, Camperdown Hb and Hb Himeji, despite evidence of uncontrolled hyperglycaemia [22, 28]. In contrast, boronate affinity chromatography and immunoassays generally produced HbA1c results consistent with glycaemic status in patients with structural haemoglobin changes, whereas ion‐exchange HPLC showed discordant results [23]. HPLC‐based HbA1c assays may be affected by haemoglobin variant interference due to coelution [33]. For discordant HbA1c results, retesting with a different analytical method is recommended. In resource‐limited settings, NGSP‐certified immunoassay‐based point‐of‐care devices may offer a practical alternative to HPLC where haemoglobin variants are prevalent.

Meanwhile, fructosamine and glycated albumin levels are unaffected by haemoglobin abnormalities [10] and retain excellent diagnostic accuracy (AUC ≈ 0.92) using cutoff values near 310–352 *μ*mol/L for diabetes [18], making fructosamine worthy of being considered a complementary biomarker for glycaemic control monitoring, especially in populations with increased prevalence of haemoglobinopathy. Glycated albumin correlated strongly with CGM mean glucose (*r* ≈ 0.84) and was unaffected by haemoglobin concentration in the OPTIMAL cohort [16]. Case reports further substantiate the clinical utility of these short‐term glycation markers. These findings support Gounden et al., indicating that fructosamine and glycated albumin are unaffected by erythrocyte‐related variables and could serve as alternatives when HbA1c is compromised [10]. Despite their clinical utility, fructosamine and glycated albumin are subject to limitations related to serum albumin concentration below 3.0 g/dL. Accordingly, clinicians should interpret these markers with caution in the context of hypoalbuminemia or nephrotic syndrome. Taken together, fructosamine and glycated albumin can accurately reflect glycaemia and detect uncontrolled DM when HbA1c is misleading, offering a practical alternative in variant carriers.

### 4.1. Clinical Implications

Our synthesis has clear practical consequences (as conceptually summarised in Figure 2). For most patients with T2DM, HbA1c remains the cornerstone of monitoring. In patients with Hb AS or Hb AC genotypes, HbA1c values can generally be interpreted reliably provided that laboratories employ NGSP‐ or IFCC‐certified immunoassays or capillary electrophoresis platforms, which exhibit minimal interference from these variants. Clinicians should continue routine HbA1c testing in these patients, but always consider other factors (anaemia and renal function) that might affect red blood cell turnover. In individuals with homozygous or compound heterozygous haemoglobin variants such as those lacking a normal HbA phenotype, HbA1c measurements should be interpreted with considerable caution. Ion‐exchange HPLC platforms are particularly prone to producing spuriously low or, in some cases, indeterminate HbA1c values in the presence of variants such as Hb E, Hb D, Hb Himeji and Hb Camperdown [33]. In such cases, HbA1c should not be used as the primary marker for glycaemic assessment. Clinicians and laboratory personnel are therefore encouraged to employ alternative assay methodologies such as boronate affinity chromatography or immunoassays and to carefully examine chromatographic profiles for atypical peaks that may indicate haemoglobin variant interference.

**Figure 2:**
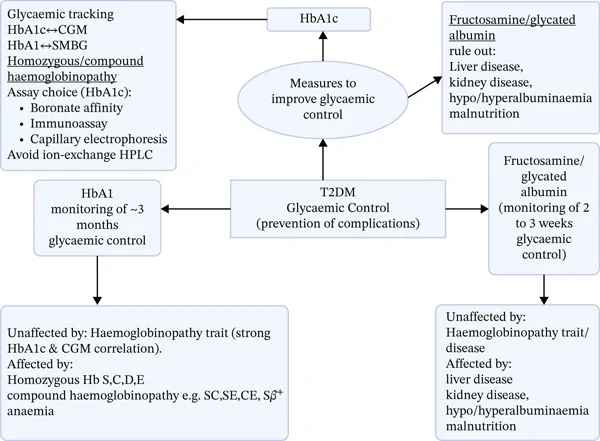
Conceptual understanding of the state of evidence regarding the impact of haemoglobinopathy on HbA1c. The figure shows a framework for selecting appropriate markers for monitoring glycaemic control in Type 2 diabetes mellitus (T2DM), demonstrating HbA1c as the primary marker for assessing glycaemic control within 3–4 months and glycated albumin or fructosamine as short‐term (2–3 weeks) alternatives. HbA1c results closely reflect blood glucose levels in T2DM individuals with no haemoglobin variants and in heterozygous traits, but may not be reliable in homozygous or compound haemoglobinopathies. Reliable assay methods such as immunoassay, boronate affinity and capillary electrophoresis are recommended, whereas ion‐exchange HPLC should be avoided in T2DM individuals with inherited haemoglobin variants. Fructosamine and glycated albumin are not affected by haemoglobin variants, making them useful alternative markers when HbA1c is unreliable, although their interpretation may be limited by abnormal albumin levels, malnutrition or liver or kidney disease. The rationale behind considering both haemoglobin‐ and protein‐based strategies is to improve the accuracy of testing so as to guide interventions that reduce T2DM‐related complications by improving glycaemic control. T2DM: Type 2 diabetes mellitus; HbA1c: glycated haemoglobin; CGM: continuous glucose monitoring; SMBG: self‐monitoring of blood glucose; Hb S/Hb C/Hb D: haemoglobin S/haemoglobin C/haemoglobin D; SC, SE, CE: compound heterozygous haemoglobin variants (e.g. Hb SC, Hb SE and Hb CE); S*β* ^+^: sickle cell *β* ^+^ thalassaemia; HPLC: high‐performance liquid chromatography. The image was created using BioRender.

When HbA1c is implausible, alternative markers such as serum fructosamine and glycated albumin can provide valuable assessments of glycaemic status (Figure 2). Serum fructosamine demonstrates a strong correlation with mean glucose levels in individuals with haemoglobin variants and can reliably identify poor glycaemic control at thresholds above 352.5 *μ*mol/L [18]. Similarly, glycated albumin accurately reflects average glucose concentrations and is unaffected by anaemia, making it a robust substitute in such contexts. In clinical practice, these assays may be requested when HbA1c results are compromised. However, interpretation requires caution as fructosamine values are dependent on serum albumin concentration and markedly reduced albumin levels (< 3 g/dL) [34] may invalidate the test. Both serum fructosamine and glycated albumin should therefore be interpreted within the broader clinical context, including assessment of liver function and nutritional status, and ideally calibrated against concurrent plasma glucose measurements or SMBG records.

Relatedly, laboratory strategies are equally important. Laboratory reports should note any identified variant and state whether the HbA1c assay used is known to be affected. Dual‐method approaches (e.g. pairing immunoassay with boronate chromatography) can help detect discrepancies early. Clinicians should be alerted if HbA1c chromatograms show abnormalities; this should trigger reflex haemoglobin electrophoresis or genetic testing. This may be particularly challenging for practitioners in low‐ and middle‐income countries (LMICs) where the routine availability of alternative assay platforms may not be feasible in the clinical setting. This may call for a renewed political will to invest in strengthening the diagnostic capacities of these LMICs′ healthcare ecosystems. It is important to note that in LMICs where haemoglobinopathies are particularly present, switching from ion‐exchange HPLC to immunoassay or POC devices for variant carriers may prevent misclassification. Implicitly, inclusion of haemoglobinopathy screening in the diagnostic workup of T2DM patients ought to be a routine clinical/laboratory undertaking, particularly in LMICs where prevalence of haemoglobinopathy is high given the evidence synthesised herein that these interfere with some HbA1c assay platforms. Overall, an interdisciplinary approach involving laboratorians, endocrinologists and geneticists is needed to manage DM in patients with haemoglobinopathies.

### 4.2. Strengths and Limitations of the Review

This review provides a synthesis of the available evidence of HbA1c and short‐term glycation markers in T2DM patients with inherited haemoglobin variants across Africa, Asia, Europe and North America. We adhered to PRISMA guidelines and employed rigorous quality assessment and GRADE evaluation to gauge evidence certainty. Nonetheless, the evidence base has constraints. Many included studies had small samples, especially the single‐site cross‐sectional analyses, yielding wide confidence intervals. Assay heterogeneity and the lack of standardised fructosamine measurements precluded formal meta‐analysis of pooled effects. Nearly all data on homozygous/compound variants come from case reports or method‐comparison papers, which carry a high risk of selection bias and confounding. Only one study provided diagnostic accuracy metrics. We also limited searches to English language publications, so some relevant data may have been missed. Finally, publication bias is likely: Unusual interference cases are more publishable than null findings. These limitations mean pooled estimates should be interpreted cautiously and underscore the low‐to‐moderate GRADE certainty for many conclusions.

### 4.3. Future Research Directions

To build on these findings, well‐designed prospective studies are needed. Multicentre longitudinal/cohort diagnostic accuracy studies should compare different HbA1c assay methods head‐to‐head (ion‐exchange HPLC, boronate affinity, immunoassay and capillary electrophoresis) against robust glycaemic references (CGM or frequent SMBG) in cohorts of patients with genotyped haemoglobin variants. Such studies should stratify by variant type (heterozygous vs. homozygous/compound), anaemia status, renal function and albumin concentration to isolate confounders. Standardisation of alternative markers is also essential: There is a need for harmonised assays and population‐specific reference ranges for serum fructosamine and glycated albumin. Once these short‐term glycaemic markers are standardised, research and development efforts to functionalise these as point‐of‐care devices will facilitate democratisation of these assay platforms across resource‐limited settings. Future work should investigate how hypoalbuminaemia, liver disease, inflammation or high protein states influence these markers in diabetes. Additionally, cost‐effectiveness and feasibility analyses are warranted, especially in low‐resource settings, to determine whether and when to deploy CGM or alternative biomarkers instead of HbA1c. Larger datasets and longitudinal designs will enable stronger evidence and clearer guidance for monitoring diabetes in the context of haemoglobinopathies.

## 5. Conclusion

HbA1c is a valuable indicator of long‐term glycaemic control for most patients with T2DM. In heterozygous haemoglobin variant carriers (e.g. Hb AS and Hb AC), assays standardised and certified by the NGSP (aligned to the IFCC reference method) [5] generally provide reliable estimates of average glucose. However, in individuals with homozygous or compound haemoglobinopathies, HbA1c values may be severely distorted or absent. In these cases, clinicians should rely on complementary measures: Serum fructosamine and glycated albumin correlate well with glycaemia in variant carriers and could offer short‐term alternatives unaffected by red blood cell kinetics. Our review highlights the need for vigilance: Confirming haemoglobin phenotype, selecting appropriate assay methods and using adjunctive markers will improve glycaemic assessment in affected populations. Although current evidence is limited, it suggests that serum fructosamine and glycated albumin hold promise for monitoring glycaemia where HbA1c is unreliable. Standardised assays and adequately powered longitudinal studies are recommended to refine these recommendations and ensure equitable diabetes care in diverse patient groups.

## Author Contributions

E.M.A. was involved in conceptualisation, methodology (database search), investigation (screening and selection, data extraction and quality assessment), data synthesis and analysis, writing the original manuscript draft, manuscript review and project administration. R.K.Y. was involved in conceptualisation and supervision, manuscript writing and review. G.N.O. was involved in conceptualisation, investigation (screening and selection, data extraction and quality assessment), data synthesis and analysis, manuscript writing and review. W.A. was involved in conceptualisation, methodology (database search), investigation (screening and selection, data extraction and quality assessment), data synthesis and analysis, manuscript writing and review. P.A. was involved in conceptualisation and supervision, methodology (protocol guarantor), project administration and manuscript review.

## Funding

No funding was received for this manuscript.

## Disclosure

All authors read and approved the final manuscript.

## Consent

The authors have nothing to report.

## Conflicts of Interest

The authors declare no conflicts of interest.

## Supporting Information

Additional supporting information can be found online in the Supporting Information section.

## Supporting information


**Supporting Information 1** File S1: The full search strategies used across Scopus, PubMed and Ovid MEDLINE, including all keywords and Boolean operators applied. File S2: The detailed quality appraisal of the included studies using the Joanna Briggs Institute (JBI) critical appraisal tools for cross‐sectional studies and case reports.

**Supporting Information 2** PRISMA 2020 Checklist

## Data Availability

Data sharing is not applicable to this article as no datasets were generated or analysed during the current study.

## References

[1] Magliano D. J. , Boyko E. J. , Genitsaridi I. , Piemonte L. , Riley P. , and Salpea P. , Diabetes Atlas, 2025, 11th edition, International Diabetes Federation, https://diabetesatlas.org/.

[2] Goyal R. , Singhal M. , and Jialal I. , Type 2 Diabetes, 2023, StatPearls Publishing, https://www.ncbi.nlm.nih.gov/books/NBK513253/. 30020625

[3] Gieroba B. , Kryska A. , and Sroka-Bartnicka A. , Type 2 Diabetes Mellitus – Conventional Therapies and Future Perspectives in Innovative Treatment, Biochemistry and Biophysics Reports. (2025) 42, 102037, 10.1016/J.BBREP.2025.102037, 40395625. PMC12090304 40395625

[4] World Health Organization , Guidance on Global Monitoring for Diabetes Prevention and Control Framework, Indicators and Application, 2024, World Health Organization.

[5] National Glycohemoglobin Standardization Program Factors That Interfere With HbA1c Test Results . In: National Glycohemoglobin Standardization Program 21 Nov 2024 [cited 20 May 2025] https://ngsp.org/factors.asp.

[6] Eyth E. , Zubair M. , and Naik R. , Hemoglobin A1c, Laboratory Screening and Diagnostic Evaluation: An Evidence-Based Approach, 2025, Springer Publishing Company, 403–408, 10.1891/9780826188434.0087.

[7] Sofronescu AG. Navigating Hemoglobin A1c Measurements for Diabetes Care|https://myadlm.org/. In: Association for Diagnostics and Laboratory Medicine [Internet]. 9 Sep 2025 [cited 2 Nov 2025] pp. 1–1. https://myadlm.org/cln/articles/2025/septemberoctober/navigating-hemoglobin-a1c-measurements-for-diabetes-care.

[8] Chen Z. , Shao L. , Jiang M. , Ba X. , Ma B. , and Zhou T. , Interpretation of HbA1c Lies at the Intersection of Analytical Methodology, Clinical Biochemistry and Hematology (Review), Experimental and Therapeutic Medicine. (2022) 24, no. 6, 10.3892/ETM.2022.11643, 36382101. PMC9634344 36382101

[9] Zhu N. A. , Reichert S. , and Harris S. B. , Limitations of Hemoglobin A1c in the Management of Type 2 Diabetes Mellitus, Canadian Family Physician. (2020) 66. PMC7021345 32060191

[10] Gounden V. , Anastasopoulou C. , Zubair M. , and Jialal I. , Clinical Utility of Fructosamine and Glycated Albumin, 2025, StatPearls Publishing, https://www.ncbi.nlm.nih.gov/books/NBK470185/. 29262081

[11] Modell B. and Darlison M. , Global Epidemiology of Haemoglobin Disorders and Derived Service Indicators, Bulletin of the World Health Organization. (2008) 86, no. 6, 480–487, 10.2471/BLT.06.036673, 18568278. PMC2647473 18568278

[12] Thomson A. M. , McHugh T. A. , Oron A. P. , Teply C. , Lonberg N. , Tella V. V. , Wilner L. B. , Fuller K. , Hagins H. , Aboagye R. G. , and Aboye M. B. , Global, Regional, and National Prevalence and Mortality Burden of Sickle Cell Disease, 2000–2021: A Systematic Analysis From the Global Burden of Disease Study 2021, Lancet Haematology. (2023) 10, no. 8, e585–e599, 10.1016/S2352-3026(23)00118-7, 37331373. PMC10390339 37331373

[13] Page M. J. , McKenzie J. E. , Bossuyt P. M. , Boutron I. , Hoffmann T. C. , Mulrow C. D. , Shamseer L. , Tetzlaff J. M. , Akl E. A. , Brennan S. E. , and Chou R. , The PRISMA 2020 Statement: An Updated Guideline for Reporting Systematic Reviews, BMJ. (2021) 372, 10.1136/BMJ.N71. PMC8005924 33782057

[14] Moiz B. , Rasool Hashmi M. , and Sadaf S. , Performance Evaluation of Ion Exchange and Affinity Chromatography for HbA1c Estimation in Diabetic Patients With HbD: A Study of 129 Samples, Clinical Biochemistry. (2008) 41, no. 14-15, 1204–1210, 10.1016/j.clinbiochem.2008.06.015. 18644359

[15] Niwaha A. J. , Rodgers L. R. , Greiner R. , Balungi P. A. , Mwebaze R. , McDonald T. J. , Hattersley A. T. , Shields B. M. , Nyirenda M. J. , and Jones A. G. , HbA1c Performs Well in Monitoring Glucose Control Even in Populations With High Prevalence of Medical Conditions That May Alter Its Reliability: The OPTIMAL Observational Multicenter Study, BMJ Open Diabetes Research & Care. (2021) 9, no. 1, e002350, 10.1136/bmjdrc-2021-002350, 34535465. PMC8451306 34535465

[16] Niwaha A. J. , Balungi P. A. , McDonald T. J. , Hattersley A. T. , Shields B. M. , Nyirenda M. J. , and Jones A. G. , Glycated Albumin and Fructosamine Do Not Improve Accuracy of Glycaemic Control Assessment in Patients With Conditions Reported to Affect HbA1 Creliability, Diabetic Medicine. (2025) 42, no. 7, e70011, 10.1111/dme.70011. 40289332

[17] Skinner S. , Diaw M. , Mbaye M. N. , Joly P. , Renoux C. , Masson C. , Cuerq C. , Lopez P. , Ndour M. , Diedhiou D. , and Sow D. , Evaluation of Agreement Between Hemoglobin A1c, Fasting Glucose, and Fructosamine in Senegalese Individuals With and Without Sickle-Cell Trait, PLoS One. (2019) 14, no. 2, 10.1371/journal.pone.0212552, 30768636. PMC6377192 30768636

[18] Doumatey A. P. , Feron H. , Ekoru K. , Zhou J. , Adeyemo A. , and Rotimi C. N. , Serum Fructosamine and Glycemic Status in the Presence of the Sickle Cell Mutation, Diabetes Research and Clinical Practice. (2021) 177, 108918, 10.1016/j.diabres.2021.108918, 34126128. PMC8447861 34126128

[19] Ama V. , Kengne A. P. , Nansseu N. J. R. , Nouthe B. , and Sobngwi E. , Would Sickle Cell Trait Influence the Metabolic Control in Sub-Saharan Individuals With Type 2 Diabetes?, Diabetic Medicine. (2012) 29, no. 9, 10.1111/j.1464-5491.2012.03620.x. 22356301

[20] Kosecki S. M. , Rodgers P. T. , and Adams M. B. , Glycemic Monitoring in Diabetics With Sickle Cell Plus *β*-Thalassemia Hemoglobinopathy, Annals of Pharmacotherapy. (2005) 39, no. 9, 1557–1560, 10.1345/aph.1G010. 16076916

[21] Thevarajah M. , Nadzimah M. N. , and Chew Y. Y. , Interference of hemoglobinA1c (HbA1c) Detection Using Ion-Exchange High Performance Liquid Chromatography (HPLC) Method by Clinically Silent Hemoglobin Variant in University Malaya Medical Centre (UMMC)-A Case Report, Clinical Biochemistry. (2009) 42, no. 4-5, 430–434, 10.1016/j.clinbiochem.2008.10.015. 19026622

[22] Castelli R. , Tempesta A. , Bianchi A. , Porro T. , Ivaldi G. , and Cappellini M. D. , Unreliable Estimation of HbA1cdue to the Presence of Camperdown Haemoglobin [*β* 104 (G6) Arg → Ser], Diabetic Medicine. (2004) 21, no. 4, 377–379, 10.1111/j.1464-5491.2004.1098.x. 15049942

[23] Yuan Y. , Zhou X. , Gao L. , Ren Q. , and Ji L. , Silent Hemoglobin Variant During Capillary Electrophoresis: A Case Report, Journal of Diabetes Investigation. (2020) 11, no. 4, 1014–1017, 10.1111/jdi.13222, 32020779. PMC7378421 32020779

[24] Sadriani N. and Marpaung F. R. , Undetectable High-Performance Liquid Chromatography Haemoglobin A1c on Variant Haemoglobin E Phenotype: A Case Report, Biochemia Medica. (2023) 33, no. 3, 10.11613/BM.2023.030801, 37841770. PMC10564149 37841770

[25] Danescu L. G. , Levy S. , and Levy J. , Markedly Low Hemoglobin A1c in a Patient With an Unusual Presentation of ß-Thalassemia Minor, Endocrine Practice. (2010) 16, no. 1, 89–92, 10.4158/EP09160.CR. 19703808

[26] Takeda Y. , Kawanami D. , and Utsunomiya K. , Detection of Hemoglobin Variant HbS on the Basis of Discrepant HbA1c Values in Different Measurement Methods, Diabetology International. (2016) 7, no. 2, 199–203, 10.1007/s13340-015-0237-8. PMC6225000 30603264

[27] Karki R. , Lamichhane S. , Jha R. , and Manandhar R. , An Incidental Finding of a Hemoglobin E Variant in a Diabetic Patient With an Abnormal Glycated Hemoglobin Level: A Case Report, Journal of Medical Case Reports. (2024) 18, no. 1, 10.1186/s13256-024-04518-y, 38877536. PMC11179220 38877536

[28] Guedes V. , Bettencourt-Silva R. , Queirós J. , Esteves M. D. L. , Teles M. J. , and Carvalho D. , Hemoglobin Himeji and Inconsistent Hemoglobin A1c Values: A Case Report, Journal of Medical Case Reports. (2017) 11, no. 1, 10.1186/s13256-017-1377-1, 28743307. PMC5527397 28743307

[29] Cheng X. , Li M. , Wu J. , and Su W. , HbG-Coushatta: An Unexpected Discovery During HbA1c Measurement, Clinica Chimica Acta. (2015) 444, 163–166, 10.1016/j.cca.2015.02.010. 25684455

[30] Yordanov E. , Hristov E. , Parvova I. , Petkova V. , and Andreevska K. , Diagnostic and Prognostic Value of Glycated Hemoglobin (HbA1c) in Patients With Diabetes Mellitus and Thalassemia, Acta Medica Bulgarica. (2020) 47, no. 3, 13–17, 10.2478/amb-2020-0027.

[31] Dholariya S. , Parchwani D. N. , Sonagra A. , Singh R. , Motiani A. , Kaliya M. , and Anandani G. , Detection of HbD Punjab Trait in Saurashtra Region of Gujarat by HPLC Method, Indian Journal of Clinical Biochemistry. (2024) 39, no. 3, 438–441, 10.1007/s12291-023-01160-1, 39005868. PMC11239634 39005868

[32] Li M. , Ge S. , Shu X. , Wu X. , Liu H. , Xu A. , and Ji L. , Interference of Hemoglobin Variants With HbA1c Measurements by Six Commonly Used HbA1c Methods, Laboratory Medicine. (2024) 55, no. 6, 708–712, 10.1093/LABMED/LMAE034. 38801245

[33] Little R. R. and Roberts W. L. , A Review of Variant Hemoglobins Interfering With Hemoglobin A1c Measurement, Journal of Diabetes Science and Technology. (2009) 3, no. 3, 446–451, 10.1177/193229680900300307. PMC2769887 20144281

[34] Baker J. R. , O′Connor J. P. , Metcalf P. A. , Lawson M. R. , and Johnson R. N. , Clinical Usefulness of Estimation of Serum Fructosamine Concentration as a Screening Test for Diabetes Mellitus, British Medical Journal (Clinical Research Ed.). (1983) 287, no. 6396), 10.1136/BMJ.287.6396.863. PMC1549287 6412861

