# Supplementary material for: Glycated Haemoglobin Versus Serum Fructosamine in Assessing Glycaemic Control Amongst Type 2 Diabetes Mellitus Patients With Haemoglobin Variants: A Systematic Review
Source: Biomed Res Int. 2026 Nov 14;2026:9298499. doi: 10.1155/bmri/9298499 (PMC13768923; doi:10.1155/bmri/9298499)
Supplement: Supplementary file 2 [file BMRI-2026-9298499-s002.docx]

**Supplementary material S1: Search strings**

**Scopus**

TITLE-ABS-KEY (“type 2 diabetes" OR T2DM) AND TITLE-ABS-KEY ("hemoglobin variant" OR "haemoglobin variant" OR "hemoglobinopathy" OR "sickle cell trait" OR "sickle cell disease" OR "beta thalassemia") AND TITLE-ABS-KEY ("HbA1c" OR "glycated hemoglobin" OR "glycosylated hemoglobin" OR fructosamine) AND TITLE-ABS-KEY ("accuracy" OR "reliability" OR "diagnostic performance" OR "glycemic control"))

**PubMed**

("Diabetes Mellitus, Type 2"[Mesh] OR "type 2 diabetes" OR T2DM)

AND ("Hemoglobinopathies"[Mesh] OR "haemoglobin variant" OR "hemoglobin variant" OR "Sickle Cell Trait"[Mesh] OR "sickle cell trait" OR "sickle cell disease" OR "beta thalassemia")

AND ("Glycated Hemoglobin A"[Mesh] OR "HbA1c" OR "glycated hemoglobin" OR "glycosylated hemoglobin" OR fructosamine)

AND ("accuracy" OR "reliability" OR "diagnostic performance" OR "glycemic control")

**MEDLINE**

1. Diabetes Mellitus, Type 2/ OR "type 2 diabetes".mp. OR T2DM.mp. 2. Hemoglobinopathies/ OR "haemoglobin variant".mp. OR "hemoglobin variant".mp. OR Sickle Cell Trait/ OR "sickle cell trait".mp. OR "sickle cell disease".mp. OR "beta thalassemia".mp. 3. Glycated Hemoglobin A/ OR HbA1c.mp. OR "glycated hemoglobin".mp. OR "glycosylated hemoglobin".mp. OR fructosamine.mp. 4. Accuracy.mp. OR reliability.mp. OR "diagnostic performance".mp. OR "glycemic control".mp. 5. 1 AND 2 AND 3 AND 4

*Supplementary material S2: JBI Assessment of the Selected Articles*

# Cross- sectional and cohort study

| Author,year (Title, Country | Inclusion criteria clear? | Subjects & setting described? | Exposure valid/reliable? | Condition measured with standard criteria? | Confounders identified? | Strategies for confounding? | Outcomes valid/reliable? | Appropriate analysis? | Overall appraisal |
| --- | --- | --- | --- | --- | --- | --- | --- | --- | --- |
| Ama et al. 2012 (SCT and HbA1c, Cameroon) | Yes | Yes | Yes | Yes | Yes | Yes | Yes | Yes | Low risk of bias |
| Skinner et al. 2019 (Agreement HbA1c vs FG vs Fructosamine, Senegal) | Yes | Yes | Yes | Yes | Yes | Unclear | Yes | Yes | Low risk of bias |
| Doumatey et al. 2021 (Serum fructosamine utility, West African cohort) | Yes | Yes | Yes | Yes | Yes | Yes | Yes | Yes | Low risk of bias |
| Niwaha et al., 2021(HbA1c performance, Uganda). | Yes | Yes | Yes | Yes | Yes | Unclear | Yes | Yes | Low risk of bias |
| Niwaha et al., 2025(Glycated Albumin and serum fructosamine do not perform better than HbA1c, Uganda). | Yes | Yes | Yes | Yes | Yes | Yes | Yes | Yes | Low risk of bias |

Abbreviations**:** JBI = Joanna Briggs Institute; Hb = haemoglobin. Notes**:** Each case report was assessed using the JBI checklist for case reports, where “Yes” indicates the criterion was met, “No” indicates it was not met, “Unclear” indicates insufficient information, and “Not applicable” indicates the criterion was not relevant, with the overall appraisal reflecting the level of risk of bias.

Case Reports

| Author, year (Hb type, Country) | Demographics? | History & Timeline? | Current Condition? | Diagnostics Clearly Described? | Intervention Described? | Post-Intervention Condition? | Adverse/  Unanticipated Events? | Takeaway Lessons? | Overall Appraisal |
| --- | --- | --- | --- | --- | --- | --- | --- | --- | --- |
| Karki et al. 2024 (HbE, Nepal) | Yes | Yes | Yes | Yes | Yes | Unclear | No/Unclear | Yes | Moderate Risk of Bias |
| Sadriani & Marpaung 2023 (HbE, Indonesia) | Yes | Yes | Yes | Yes | Yes | Unclear | No | Yes | Moderate Risk of Bias |
| Yordanov et al. 2020 (Thalassemia, Bulgaria) | Yes | Yes | Yes | Yes | Yes | Unclear | No | Yes | Moderate Risk of Bias |
| Kosecki et al. 2005 (Sickle + β-thalassemia, USA) | Yes | Yes | Yes | Yes | Yes | Yes | No | Yes | Moderate Risk of Bias |
| Takeda et al. 2015 (HbS, Japan) | Yes | Yes | Yes | Yes | Yes | Unclear | No | Yes | Moderate Risk of Bias |
| Yuan et al. 2020 (Hb North Manchester, China) | Yes | Yes | Yes | Yes | Yes | Unclear | No | Yes | Moderate Risk of Bias |
| Thevarajah et al. 2009 (HbD Punjab, Malaysia) | Yes | Yes | Yes | Yes | Yes | Unclear | No | Yes | Moderate Risk of Bias |
| Cheng et al. 2015 (HbG-Coushatta, China) | Yes | Yes | Yes | Yes | Yes | Unclear | No | Yes | Moderate Risk of Bias |
| Danescu et al. 2010 (β-thalassemia minor, USA) | Yes | Yes | Yes | Yes | Yes | Unclear | No | Yes | Moderate Risk of Bias |
| Dholariya et al. 2023 (HbD Punjab, India) | Yes | Yes | Yes | Yes | Yes | Unclear | No | Yes | Moderate Risk of Bias |
| Guedes et al. 2017  (Hb Himeji, Portugal) | Yes | Yes | Yes | Yes | Yes | Unclear | No | Yes | Moderate Risk of Bias |
| Castelli et al., 2024(β104 (G6) Arg→Ser, Italy). | Yes | Yes | Yes | Yes | Yes | No | Not Applicable | Yes | Moderate Risk of Bias |

Abbreviations**:** JBI = Joanna Briggs Institute; Hb = haemoglobin. Notes**:** Each case report was assessed using the JBI checklist for case reports, where “Yes” indicates the criterion was met, “No” indicates it was not met, “Unclear” indicates insufficient information, and “Not applicable” indicates the criterion was not relevant, with the overall appraisal reflecting the level of risk of bias.
